# Survey of the knowledge, attitude and perceptions on bovine tuberculosis in Mnisi community, Mpumalanga, South Africa

**DOI:** 10.4102/ojvr.v87i1.1808

**Published:** 2020-07-30

**Authors:** Rudo Marange, Darshana Morar-Leather, Folorunso O. Fasina

**Affiliations:** 1 Department of Veterinary Tropical Diseases, Faculty of Veterinary Science, University of Pretoria, Onderstepoort, Pretoria, South Africa; 2 Food and Agriculture Organization of the United Nations, Dar es Salaam, United Republic of Tanzania

**Keywords:** bovine tuberculosis, awareness, Mpumalanga, surveillance, zoonosis

## Abstract

Tuberculosis (TB) is a global health concern of zoonotic importance, and *Mycobacterium bovis* and *Mycobacterium tuberculosis* are the most common causes of TB in animals and humans, respectively. Integral to TB control strategies are the communities affected by this epidemic. Tuberculosis awareness by the community is an effective TB control strategy as education empowers people to make informed choices with regard to mitigating TB risk factors in their daily lives. We conducted a knowledge, attitude and perceptions survey in Mnisi pastoral community in South Africa using a semi-structured questionnaire to evaluate the level of bovine TB (bTB) awareness, and provided informed feedback to the community on the outcome of the study. Although participants were aware of TB, the knowledge of the zoonotic potential of bTB and about susceptible hosts was limited. The study findings showed knowledge gaps regarding common risk factors, including coughing while herding cattle, unsupervised/uninspected communal slaughter and improper disposal of infected meat. In contrast, it was noted that the majority of participants discarded meat with visible lesions and consumed pasteurised milk; thus, the risk of TB transmission via the ingestion route is low. Tuberculosis knowledge gaps were evident in the community, and public health and veterinary authorities need to improve relationships with stakeholders and implement awareness programmes that use a one health approach.

## Introduction

*Mycobacterium bovis* is the causal agent of bovine tuberculosis (bTB), an infectious and chronic disease in animals and humans (Neill, Skuce & Pollock 2005). *Mycobacterium bovis* threatens wildlife conservation and the economy, and zoonotic TB appears aggravated in developing countries because of the poor veterinary infrastructures, paucity of funds for disease prevention and controls (Etter et al. 2006; Olea-Popelka et al. 2017). In low-to-middle-income countries (LMICs) in Africa, bTB is prevalent in cattle populations (Ayele et al. 2004) and impacts animal production negatively (e.g. carcass condemnations, decreased milk yields, low meat yield because of emaciation and poor reproductive performances). Such production losses or a single animal death has far-reaching economic and sociocultural consequences on subsistence farmers in resource-poor communities (Olea-Popelka et al. 2017).

Bovine TB has been implicated in the increasing cases of human health problems in Africa, such as the human immunodeficiency virus (HIV) pandemic (Cosivi et al. 1998), and an increased human TB because of *Mycobacterium tuberculosis* may suggest a concomitant increase in human infections with *M. bovis*. In LMICs, human–animal interactions are intensive, with close cohabitation and poor hygiene standards during handling of animal products (Ameni et al. 2007).

While drinking raw milk and eating undercooked meat are obvious risk factors for the transmission, traditional practices such as sharing of dwellings with animals at night and plastering houses with cow dung are inadvertent risk for bTB transmission (Katale et al. 2012).

Addressing the identified risk factors is sometimes complicated by cultural resistance (Olea-Popelka et al. 2017). Attempting to change norms, such as plastering houses with cow dung, without giving cheaper more accessible alternatives would be met with resistance. For example, suggesting cement as an alternative building material comes with high cost and is not a feasible alternative to the already impoverished communities. Because livestock disease monitoring, control and eradication activities should use multi-partner, multi-disciplinary approaches and should be farmer-centred, policy-makers should take cognisance of farmers’ awareness levels and perceptions on bTB in planning mitigations and control strategies (Ameni et al. 2007).

## Materials and methods

Mnisi is located in the north-eastern corner of Bushbuckridge Municipal Area of Mpumalanga province of South Africa (24.8398°S, 31.0464°E), with approximately 12 832 cattle and 15 diptanks (Musoke et al. 2015). Based on the records from the Mpumalanga Veterinary Service, a total of 1447 livestock farmers were identified and divided amongst the 15 diptanks. Using a simple ballot system, five of the diptanks were selected. Sample size was calculated using the following formula:

sample size

n=[DEFF*Np(1−p)]/[(d2/Z21−α/2*(N−1)+p*(1−p)], [Eqn 1]

Where population size (for finite population correction factor – fpc) (N): 552 farmers; hypothesised % frequency of outcome factor in the population (p): 50% ±5; confidence limits as % of 100(absolute ±%)(d): 5%; design effect (for cluster surveys-DEFF): 1. Using proportional representations, 127 farmers were recruited for questionnaire administration (minimum of 20 and maximum of 30 farmers per diptank’s catchment area). All questions were prepared in English and translated into Xitsonga, the lingua franca of the community. Data were captured, filtered and analysed for descriptive statistics using Microsoft Excel^®^.

### Ethical consideration

The project was approved by the University of Pretoria Animal Ethics Committee (AEC) (certificate number v116-16) and the Faculty of Health Sciences Research Ethics Committee (certificate number 374/2016). Informed consent was obtained from all the participants who were briefed about their right to withdraw from the study if they wished so and guaranteed confidentiality.

## Results and discussion

A total of 110 participants completed questionnaire (Table 1). Approximately 94% of participants were aware of TB. Mpumalanga has the second highest HIV prevalence in South Africa at 35.1% (Zuurendonk 2014), and the level of awareness could probably be because of the HIV/AIDS and TB awareness programmes and mass media campaigns. However, disease-specific knowledge was not matched with awareness level. As 61% of the participants associated TB with humans only, it is inferred that many are unaware of its zoonotic potential. This gap creates an inadvertent risk of zoonotic infection to humans who live in close contact and interact with cattle and goats. In Ethiopia, 84% of studied participants were unaware of transmission routes and risk factors associated with TB (Tschopp et al. 2015). Similar studies in India revealed that poor communities that have high TB prevalences with little exposure to mass media also have little knowledge of factors affecting TB risk and transmission (Sreeramareddy, Harsha Kumar & Arokiasamy 2013).

**TABLE 1:** Mnisi community tuberculosis questionnaire results.

Variable (*n* = 110)	Category	Percentage of participants
Gender	Male	64.2
	Female	35.8
Heard of TB	Yes	93.6
	No	6.4
TB hosts	Don’t know	1.8
	Humans only	60.9
	Cattle and goats	2.7
	Cattle and humans	13.6
	Humans, cattle and goats	20.9
TB diagnosed in household	Yes	21.8
	No	78.2
Herd cattle when coughing	Yes	54.5
	No	45.5
Goats and cattle herded together	Yes	34.5
	No	65.5
Source of milk	Commercially prepared milk (CPM)	83.6
	CPM and raw milk	13.6
	raw milk only	2.7
Concerned on consuming diseased animals	Yes	84.4
	No	15.6
Willingness to call vet services	Yes	88.2
	No	11.2
Willingness to offer sample	Free	92.7
	At a fee	6.4
	Not willing	< 1.0
Frequency of coming to diptank currently	Weekly	97.3
	Everyday	0.9
	Monthly	0.9
	Never	0.9
How often they would like to come to diptank for other procedures excluding dipping	Weekly	44.0
	Monthly	42.0
	Yearly	11.0
	Never	6.0
Communal slaughtering	Yes	99.1
	No	0.9
Fate of slaughtered meat	Consumed by household	28.2
	Shared with friends, relatives	65.5
	Sold	6.3
Seen TB like lesions before	Yes	91.7
	No	8.3
Measures taken when lightly infected meat seen	Ignore and cook	2.7
	Cut out infected piece	26.3
	Throw out whole organ	32.8
	Throw out whole carcase	38.2
Measures taken when grossly infected meat seen	Ignore and cook	0.0
	Cut out infected piece	11.0
	Throw out whole organ	39.0
	Throw out whole carcass	50.0
Disposal of infected part	Bin	1.8
	Bury or burn	80.0
	Feed to dogs	17.3
	Other	0.9
Refrigerators available in household	Yes	99.1
	No	< 1.0

TB, tuberculosis; CPM, commercially prepared milk.

More than half of the participants in our study would herd animals while coughing persistently. A proportion of the human population can be asymptomatic carriers of *M. bovis* (Green 2016), and many cough cases in rural populace go uninvestigated unless they become life threatening (Matebesi, Meulemans & Timmerman 2005). In addition, human origin *M. tuberculosis* has been isolated in cow’s milk, indicating the probable human–cattle transmission (Regassa, Medhin & Ameni 2008). Targeted TB awareness campaigns on the knowledge of transmission and risks to promote behavioural changes that discourage such occupationally risky behaviour will be pertinent to promote healthcare-seeking behaviour (Sreeramareddy et al. 2013).

Only 34% of the respondents herded cattle and goats together. Low inter-herd risk of disease transmission between goats and cattle has been confirmed in the pastoralist communities of Ethiopia (Tschopp et al. 2015). Communal slaughter remains a notable risk factor for plausible spread of zoonotic TB. Ninety-nine per cent of the community members confirmed to have carried out communal slaughter without the supervision of any veterinary health officials, and approximately 28% will consume the meat at home with family members, while more than 70% of them even share the meat with the public. With such practices, a single case of TB in a slaughtered animal or any other rapidly spreading infectious disease like anthrax has the potential of reaching to a significant proportion of the community and may exacerbate communal TB spread. Approximately 92% of the participants have seen TB-like lesions before in slaughtered animals, and almost 100% are at risk of infection through sharing, purchase and home consumption of uninspected meat. Given that communal slaughter might continue for the foreseeable future because of poverty, salvage slaughter, inability of rural farmers to compete fairly in the formal markets and lesser intensity of veterinary service delivery in rural areas, incentivised self-reporting should be promoted with additional benefits of improved case reporting and reduced burden of zoonoses.

The frequency of slaughter per household in this survey is however low. Typically, in South Africa and other developing countries, a reluctance to slaughter large ruminants is linked to wealth preservation based on heads of cattle owned (Green 2016). Only 2.7% of participants would ignore and cook meat with TB-like lesions. This affirmed earlier findings, which stated that most farmers in the Mnisi community discard meat with visible abnormalities (Musoke 2016). It is noteworthy that the majority of the participants understood the dangers of eating meat with apparent lesions, yet only 38% of respondents would throw out the whole carcass. Of the respondents that will dispose the potentially infected carcasses, 20% opted for discarding in bins or feeding the meat to the dogs, a method that significantly increases the risk of exposure and communal transmission of *M. bovis*. Scavengers and carnivores can contract TB from meat sources and from recycled environmental contamination and human sources (Millán et al. 2008; Moravkova et al. 2011; Thoen et al. 2009). The risk of acquired infection from drinking infected raw milk in the Mnisi community is low as only 2.7% of the participants drink raw milk irregularly. This contradicts popular belief that people in rural areas in LMICs are at risk of contracting *M. bovis* through drinking of unpasteurised milk (ingestion route) (Musoke 2016). The reasons for this good practice were not ascertained in this study but the ready accessibility of pasteurised milk, powdered milk and coffee creamers in petty trading stores, *tuckshops* or *spaza shops*, may be linked to this observation.

The respondents suggested to improve veterinary services through the provision of more facilities and extensive repairs of current infrastructure. Certain individuals displayed a clear disconnect and some level of mistrust between the community and veterinary services. Such opinions or perceptions include top-down approach to policy formulation or implementation, veterinary officials sometimes carry out procedures on livestock without explaining the basis and purpose of the procedures to owners and lack of adequate feedback from earlier studies conducted in the community. In Britain, breakdown in relationships between the state authorities and farmers was linked with differential farmer attitudes and views to most effective scientifically based interventions and the practicality of state recommendations on bTB (Cowie et al. 2014). Such discordance will negatively impact the implementation of and compliance with recommendations on eradication. The multiple roles of state authorities, such as policy-makers, enforcers and advisors, to farmers on various issues sometimes put them in conflicting positions; notwithstanding, efforts must be intensified to create harmony and mutual trust amongst all stakeholders for bTB control. In this instance, a feedback session was held to educate farmers about TB knowledge gaps identified during the study. The farmers appreciated the good practice of providing feedback to communities where studies are conducted and requested more actions to cultivate trust between researchers and farmers.
